# Can Magnetic Resonance Imaging Replace Mammography and Ultrasonography for the Detection of Breast Lesions?

**DOI:** 10.7759/cureus.8087

**Published:** 2020-05-13

**Authors:** Yeliz Yilmaz, Gulten Sezgin Bener, Kemal Atahan, Nihan Acar, Turan Acar, Haldun Kar, Furkan Tosun, Melek Gokova

**Affiliations:** 1 General Surgery, İzmir Katip Celebi University, Atatürk Training and Research Hospital, İzmir, TUR; 2 Radiology, İzmir Katip Celebi University, Atatürk Training and Research Hospital, İzmir, TUR

**Keywords:** breast tumor, imaging, mammography, ultrasonography, bi-rads, magnetic resonance imaging

## Abstract

Objective

We aimed to evaluate the role of magnetic resonance imaging (MRI) in the visualization of breast lesions and to estimate whether MRI can be a reliable alternative to mammography (MG) and ultrasonography (USG) for this purpose.

Materials and methods

In this retrospective, single-center study, an analysis of medical files of 260 patients with breast masses as breast imaging reports and data system (BI-RADS) 4 and 5 at MRI was performed. The features of the breast lump, such as the side, location, multi foci or multicentricity, histopathological diagnosis, contrast-enhancement characteristics, radiological, and pathological axillary involvement, were noted. Consistency between MRI-BIRADS and MG+USG-BIRADS, as well as the association between lesion characteristics, was sought.

Results

The agreement ratio between the BI-RADS categories of MRI and MG+USG was 0.654 while consistency between histopathological diagnosis and MRI BI-RADS category was 0.838. The agreement between the BI-RADS category of MG+USG and histopathological diagnosis was 0.819. The consistency between MRI BI-RADS and MG+USG BI-RADS increased remarkably with the advancement of age. Similarly, the consistency between MRI BI-RADS and histopathological diagnosis tends to increase with the advancement of age. Nonmass contrast enhancement yielded the highest agreement ratios between MRI BI-RADS and MG+USG BI-RADS, histopathological diagnosis and MRI BI-RADS, and histopathological diagnosis and MG+USG BI-RADS.

Conclusion

Dynamic MRI is a useful and reliable method for imaging breast neoplasms. However, it is not devoid of disadvantages such as cost, attainability, and contrast use and it should be reserved as a problem-solving technique to be used in conjunction with conventional methods including MG and USG.

## Introduction

Breast cancer constitutes one of the most important health problems in women, and survival depends on the size of the tumor and the involvement of lymph nodes at the time of diagnosis. Imaging of the breast tissue aims to screen asymptomatic cases and to evaluate the symptomatic patients properly. Since early diagnosis is the most crucial prognostic parameter, screening modalities have gained significance [1].

Even though the primary screening modality is mammography (MG), the accuracy of this method is reduced remarkably in dense breast tissues in the pre-menopausal period and cases receiving hormone replacement treatment. Moreover, MG may fail to distinguish benign tumors from malignant lesions attributed to its low specificity [2-3].

Ultrasonography (USG) constitutes an alternative diagnostic tool, particularly for patients with obscure imaging features under MG. The conjunctive use of MG and USG may not be sufficient to identify and document the behavioral patterns, multicentricity, and planning of conservative surgery and distinguish a residual lesion from granulation tissue. In such circumstances, magnetic resonance imaging (MRI) can be a useful diagnostic measure [4-5]. Owing to the use of contrast agents, MRI became a highly sensitive imaging modality for the screening and diagnosis of breast tumors. The sensitivity and specificity of MRI were reported as 83%-99%, and 87%-97%, respectively [6-9].

The philosophy of MRI is based on the assessment of longitudinal relaxation time (T1), transverse relaxation time (T2), and hydrogen spin density of tissues under investigation. There has been controversy on whether a breast lesion with contrast enhancement on MRI should be evaluated with high spatial resolution or dynamic contrast views should be preferred to achieve higher sensitivity [10]. Currently, attributed to the development of the MRI gradient system and pulse sequences, the concomitant use of high spatial resolution and sufficient temporal resolution has been popularized [10].

Increased awareness and a better understanding of the pathologic features of breast tumors may be useful to interpret the MRIs. Large lesional size (>10 mm), ill-defined margin, and irregular outline are mostly consistent with malignancy. These correlate with the pathological features of a breast tumor, characterized by the rapid growth rate, large size, infiltrative growth pattern, invasion into stroma resulting in desmoplasia, and, hence, irregular outline and margin. An integrated evaluation of clinical, radiological, and pathological characteristics is mandatory to accomplish highly accurate diagnostic outcomes [11]. The morphology and enhancement kinetics are helpful for the recognition of malignant breast tumors. In addition to discrimination of malignant and benign lesions, MRI can provide advantages for the detection of axillary lymph node metastases and the identification of occult primary [12].

The most commonly used reporting and classification system is Breast Imaging Reporting and Data System (BI-RADS®), which was used with high-resolution 3 T systems [13-14]. BI-RADS categorization has been employed for the breast lesions using the morphological features and time-signal contrast curves of the lump. Dynamic MRI is useful for the BI-RADS classification of the breast lesions and to foresee the histopathological diagnosis. Dynamic contrast-enhanced MRI constitutes an improved imaging modality with advanced accuracy for differentiation between benign and malignant lesions. Early enhancement is supposed to imply malignancy [15-16].

The present study aimed to evaluate the role of MRI in the visualization of breast lesions and to estimate whether MRI can be a reliable alternative to MG and USG for this purpose.

## Materials and methods

Study design

This retrospective, single-center study was carried out in the radiology department of a tertiary care center between January 2014 and July 2019. The approval of the local institutional review board was obtained before the study (date: 01/2020 - no:530). The study was conducted in compliance with the principles of the Helsinki Declaration.

The descriptive and radiologic information was extracted from the medical files of our hospital database. Patients with breast lesions who were classified as BI-RADS 4 and 5 on MRI views were included. The consistency of the pathological lesions with the lesion type, contrast enhancement patterns, and radiological features on MRI was investigated. The harmony between BI-RADS classification and pathological diagnosis, as well as the accuracy for the demonstration of the axillary metastasis using MRI, were assessed. Patients who underwent both MG+USG and MRI for breast lesions were included in this study. All MRI views were evaluated by the same radiologist.

Data were collected from the medical records of 260 patients (258 women, 2 men) with an average age of 50.88±11.61 (range: 20 to 80).

Outcome measures

Baseline descriptions, such as age and gender, BI-RADS classifications as for MG+USG and MRI, features of the breast lump, such as the side, location, multi foci or multicentricity, histopathological diagnosis, contrast-enhancement characteristics, and radiological and pathological axillary involvement, were noted. The location of the lesion was classified as the upper outer quadrant, upper inner quadrant, lower outer quadrant, lower inner quadrant, and central. The side of the lesion was classified as right, left, or bilateral.

Conventional diagnostic imaging

All patients underwent digital MG (IMS Giotto, Italy) and targeted ultrasonographic examination of the affected breast and the ipsilateral axillary region. Ezu-MT28-S1 model (Hitachi Inc. Japan) and a 13 MHz linear transducer were used to evaluate the breast lesions before MRI. These examinations were performed by our radiology department in our tertiary care center.

MRI protocol

Magnetic resonance imaging examinations were carried out using a 1.5-T imaging uni (Signa, GE Healthcare, Chicago, Illinois). For the Signa scanner, the imaging parameters were as follows: 4.6/2.2; ﬂip angle, 10°; a field of view (FOV), 34 × 34 cm; matrix, 320 × 320; section thickness, 2 mm; and acquisition time, 75 seconds. Non-contrast T2 and T1-weighted images were obtained in the prone position using the breast coil. A 0.01 mmol/kg contrast agent was used for contrast imaging (gadoterate meglumine Dotarem®️, Guerbet; gadobutrol: Gadovist®️, Bayer Healthcare) at a rate of 2 mL/s, which was followed by a 20 mL saline flush at the rate of 2 mL/s). Dynamic contrast-enhanced images were obtained at minutes one, two, and six after contrast material injection. Post-processing manipulation included the production of standard subtraction and maximum-intensity-projection images (MIP). The images were transferred to a workstation (Advantage Windows, software version 4.0, GE Healthcare) for analysis.

## Results

The descriptive features of our series are presented in Table 1. Our patient population (n=260) comprised 258 women and two men. The average age was 50.88±11.61 (range: 20 to 80). On MRI views, 92 lesions (35.4%) were reported as suspicious while 168 lumps were identified as highly probably malignant. The lesions most commonly involved the left side (n=132, 50.8%), followed by right side (n=113, 43.5%) and bilateral involvement (n=15, 5.8%). The number of multifocal and multicentric lesions were 40 (15.4%) and 35 (13.5%), respectively.

**Table 1:** Descriptive data (n=260) MRI - magnetic resonance imaging; MG - mammography; USG - ultrasonography BI-RADS®: Breast Imaging Reporting and Data System

		n	%
Sex	Male	2	0.8
	Female	258	99.2
MRI	Suspicious	92	35.4
	Highly probably malignant	168	64.6
Site of lesion	Right	113	43.5
	Left	132	50.8
	Bilateral	15	5.8
Location of lesion	Upper outer	108	41.5
	Upper inner	39	15.0
	Lower outer	43	16.5
	Lower inner	20	7.7
	Central	50	19.2
Multi foci	Yes	40	15.4
	No	220	84.6
Multicentricity	Yes	35	13.5
	No	225	86.5
BI-RADS for MG+USG/MRI	Additional investigation needed	71	27.3
	Negative	3	1.2
	Benign	10	3.8
	Probably benign	15	5.8
	Suspicious	47	18.1
	Highly probably malignant	78	30.0
	Proven malignancy	36	13.8
Histopathology	Benign	42	16.2
	Premalignant	207	79.6
	Malignant	11	4.2
Lesion type	Mass lesion with contrast enhancement	193	74.2
	Contrast enhancement without mass lesion	62	23.8
	Focus	5	2
Axillary involvement on MRI	Yes	126	48.5
	No	134	51.5
Pathological axillary involvement	Yes	85	32.7
	No	121	46.5
	Undetermined due to lack of surgical procedure	54	20.8

The assessment of breast MRI was performed by the same radiologist and the report included the indication for scan and clinical information in addition to the dose and type of contrast material administered to the patient. The image findings under foci were breast density, the amount of parenchymal background enhancement, and relevant findings such as axillary involvement and side and location of the lesion. Even though lymph node evaluation is not a particular aim of breast MRI, it can reveal unsuspected axillary nodal involvement. Each report was associated with a diagnostic category and recommendations. The most commonly used reporting and classification system is BI-RADS [13-14].

This system that provides the convenience of communication between clinic-radiology, as well as standardization of the studies, was offered by the American College of Radiology (ACR) in 1993 for the standardization of MG reporting terminology in the name BI-RADS [17]. This internationally accepted system was reviewed in 2003 with the addition of the MG+USG and MRI classification [18]. The BI-RADS diagnostic categories are demonstrated in Table 2.

**Table 2:** BI-RADS diagnostic categories BI-RADS®: Breast Imaging Reporting and Data System

Category	Definition
0	Incomplete, additional imaging evaluation is needed
1	Negative, no abnormalities
2	Benign findings
3	Probably benign findings
4	Suspected malignancy
5	Highly suspected malignancy
6	Already histologically proven cancer

**Figure 1:**
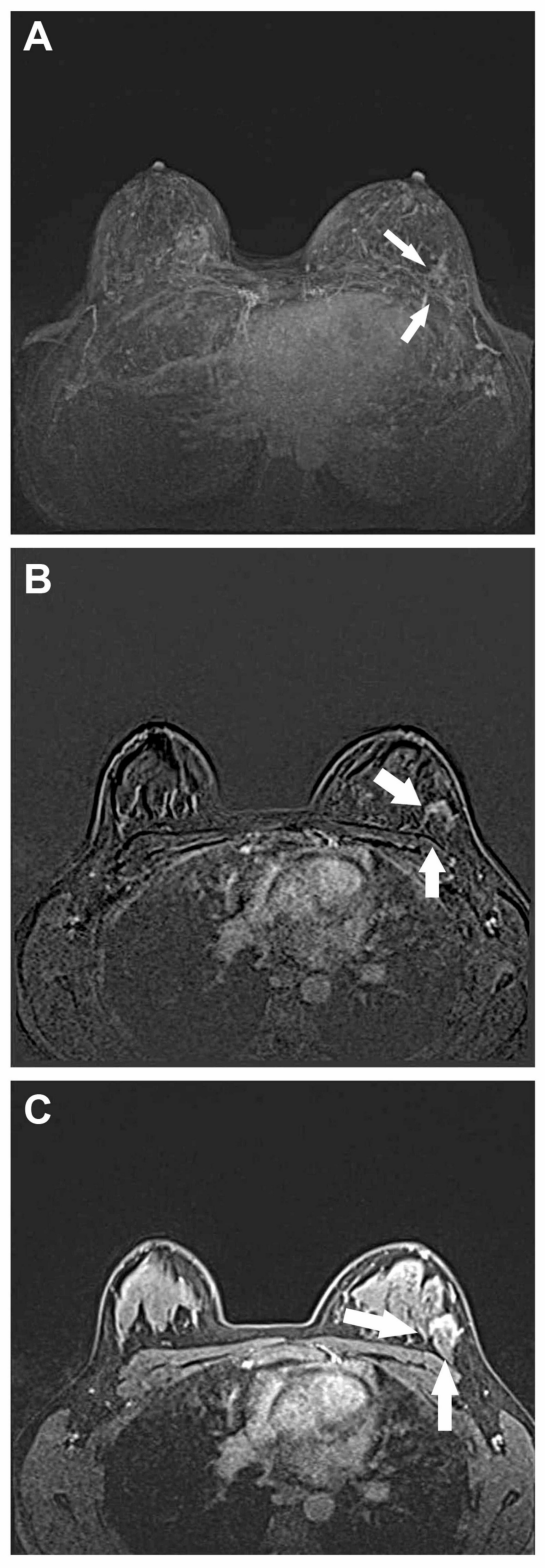
Benign sclerosing adenoma lesion Nonmass enhancement in the upper outer quadrant in the left breast was detected on maximum intensity projection (a), on the subtracted image (b), and on the first passage of dynamic enhancement images (c). The lesion was consistent with sclerosing adenosis.

**Figure 2:**
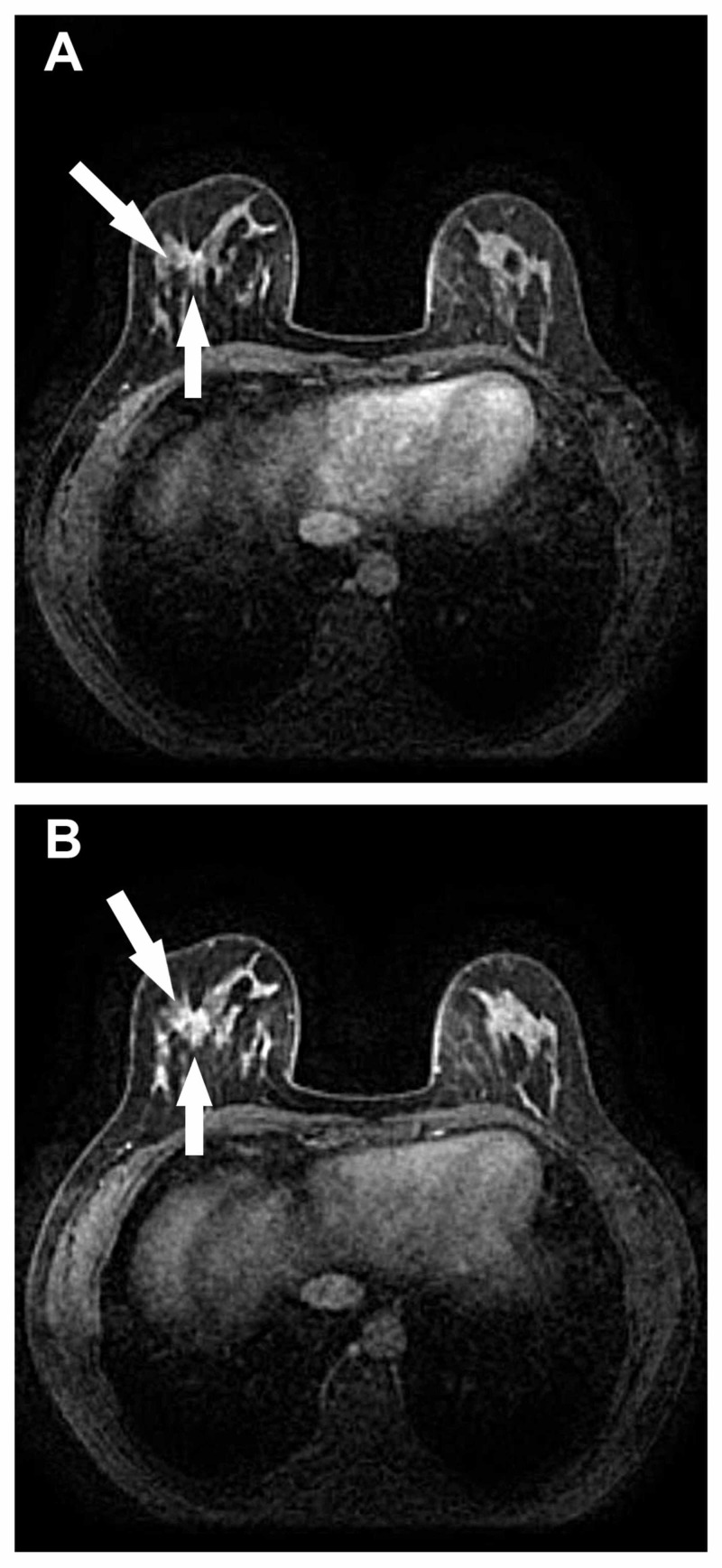
Benign sclerosing adenoma lesion A structural distortion area was detected in the lower outer quadrant of the left breast on the second (a) and sixth passages of dynamic enhancement images (b). The lesion was sclerosing adenosis.

Figures 1-2 demonstrate two benign sclerosing adenoma lesions, which were false-positively diagnosed as BI-RADS 5 at MRI. Thus, it must be remembered that despite its high sensitivity and acceptable specificity, a substantial rate of false positive and false negative results are likely particularly if MRI is used alone.

In Table 3, the agreement between the BI-RADS classification as for MRI and MG+USG and the relationship between the MRI BI-RADS category and other parameters under investigation were sought. The agreement ratio between the BI-RADS categories of MRI and MG+USG was 0.654 while consistency between histopathological diagnosis and MRI BI-RADS category was 0.838. The agreement between the BI-RADS category of MG+USG and histopathological diagnosis was 0.819.

**Table 3:** Agreement ratios between MRI BI-RADS, MG + USG BI-RADS, axillary lymph node involvement on MRI and histopathological diagnosis MRI: magnetic resonance imaging; MG: mammography, USG: ultrasonography; BI-RADS®: Breast Imaging Reporting and Data System

Variables	Agreement	Disagreement	Total	Agreement ratio
MRI BI-RADS-MG+USG BIRADS	170	90	260	0.654
MRI BI-RADS-Histopathological diagnosis	218	42	260	0.838
MG+USG BI-RADS-Histopathological diagnosis	213	47	260	0.819
MRI axillary lymph node-Histopathological diagnosis (specificity)	180	80	260	0.692
MRI axillary lymph node-Histopathological diagnosis (sensitivity)	176	84	260	0.677

Table 4 outlines the agreement ratios between MRI BI-RADS, MG+USG BI-RADS, and histopathological diagnosis as for various radiological characteristics on dynamic MRI views. We observed that the consistency between MRI BI-RADS and MG+USG BI-RADS increased remarkably with the advancement of age. Similarly, the consistency between MRI BI-RADS and histopathological diagnosis tends to increase with the advancement of age. Nonmass enhancement yielded the highest agreement ratios between MRI BI-RADS and MG+USG BI-RADS, histopathological diagnosis and MRI BI-RADS, and histopathological diagnosis and MG+USG BI-RADS.

**Table 4:** The agreement ratios between MRI BI-RADS, MG+USG BI-RADS and histopathological diagnosis as for various radiological characteristics on dynamic MRI views MRI: magnetic resonance imaging; MG: mammography; USG: ultrasonography; BI-RADS®: Breast Imaging Reporting and Data System

MRI BI-RADS & MG+USG BI-RADS	Agreement	Disagreement	Total	Agreement ratio
Age groups				
0-20	1	0	1	1.000
21-40	33	9	42	0.786
41-60	106	58	164	0.646
>60	30	23	53	0.566
MRI BI-RADS & Histopathological Diagnosis				
Age groups				
0-20	1	0	1	1.000
21-40	39	3	42	0.923
41-60	139	25	164	0.820
>60	39	14	53	0.641
MG+USG BI-RADS & Histopathological diagnosis				
Age groups				
0-20	1	0	1	1.000
21-40	34	8	42	0.810
41-60	135	29	164	0.823
>60	44	9	53	0.830
MRI BI-RADS & MG+USG BI-RADS				
Side				
Right	35	7	113	0.690
Left	84	48	132	0.636
Bilateral	8	7	15	0.533
MRI BI-RADS & Histopathological diagnosis				
Side				
Right	93	20	113	0.823
Left	110	22	132	0.833
Bilateral	15	0	15	1.000
MG+USG BI-RADS & Histopathological diagnosis				
Side				
Right	92	21	113	0.814
Left	110	22	132	0.833
Bilateral	11	4	15	0.733
MRI BI-RADS & MG+USG BIRADS				
Location of neoplasm				
Upper outer quadrant	70	38	108	0.648
Upper inner quadrant	24	15	39	0.615
Lower outer quadrant	34	9	43	0.791
Lower inner quadrant	11	9	20	0.550
Central	31	19	50	0.620
MRI BI-RADS & Histopathological diagnosis				
Location of neoplasm				
Upper outer quadrant	91	17	108	0.843
Upper inner quadrant	29	10	39	0.744
Lower outer quadrant	37	6	43	0.860
Lower inner quadrant	18	2	20	0.900
Central	43	7	50	0.860
MG+USG BI-RADS & Histopathological diagnosis				
Location of neoplasm				
Upper outer quadrant	91	17	108	0.843
Upper inner quadrant	32	7	39	0.821
Lower outer quadrant	33	10	43	0.767
Lower inner quadrant	15	5	20	0.750
Central	42	8	50	0.840
MRI BI-RADS & MG+USG BI-RADS				
Multifocal				
No	144	76	220	0.655
Yes	26	14	40	0.650
MRI BI-RADS & Histopathological diagnosis				
Multifocal				
No	182	38	220	0.827
Yes	36	4	40	0.900
MG+USG BI-RADS & Histopathological diagnosis				
Multifocal				
No	181	39	220	0.823
Yes	32	8	40	0.800
MRI BI-RADS & MG+USG BI-RADS				
Multicentricity				
No	145	80	225	0.644
Yes	25	10	35	0.714
MRI BI-RADS & Histopathological diagnosis				
Multicentricity				
No	188	37	225	0.836
Yes	30	5	35	0.857
MG+USG BI-RADS & Histopathological diagnosis				
Multicentricity				
No	184	41	225	0.818
Yes	29	6	35	0.829
MRI BI-RADS & MG+USG BI-RADS				
Type of lesion				
Contrast enhanced mass	122	71	193	0.632
Contrast enhancement without mass	46	16	62	0.742
Focal	1	1	2	0.500
MRI BI-RADS &Histopathological diagnosis				
Type of lesion				
Contrast enhanced mass	168	25	193	0.870
Contrast enhancement without mass	45	17	62	0.726
Focal	2	0	2	1.000
MG+USG BI-RADS &Histopathological diagnosis				
Type of lesion				
Contrast enhanced mass	162	31	193	0.839
Contrast enhancement without mass	46	16	62	0.742
Focal	2	0	2	1.000
MRI BI-RADS & MG+USG BI-RADS				
Contrast enhancement				
Fast	89	41	130	0.685
Slow	81	49	130	0.623
MRI BI-RADS &Histopathological diagnosis				
Contrast enhancement				
Fast	108	22	130	0.831
Slow	110	20	130	0.846
MG+USG BI-RADS &Histopathological diagnosis				
Contrast enhancement				
Fast	112	18	130	0.862
Slow	101	29	130	0.777

## Discussion

We aimed to investigate the role of MRI in imaging breast lesions and in seeking the impacts of descriptive, clinical, and radiological variables on the agreement between MRI, MG+USG, and histopathological diagnosis. Our results demonstrated that the age of the patients, the location of the lesion, and enhancement characteristics might influence the ratio of agreement between MRI and MG+USG for the detection of breast neoplasms. Therefore, complementary use of imaging modalities together with an integrative analysis of clinical, pathological, and radiological data is mandatory to improve early and accurate diagnosis rates.

Magnetic resonance imaging is an important diagnostic measure in breast imaging, and dynamic contrast-enhanced MRI is the backbone of any breast MRI protocol, with excellent sensitivity and good specificity for breast cancer diagnosis. It yields high-resolution morphological information and functional data about angioneogenesis as a tumor-specific feature [19].

The superiority of MRI is linked with its high sensitivity in tumor detection owing to the consistent contrast enhancement of breast cancer. In breast cancer, the enhancement of the tumor is always stronger than the normal breast tissue. Lack of enhancement reminds a benign lesion or normal tissue. Even pre-invasive lesions, such as lobular carcinoma in situ, showed stronger enhancement than normal glandular tissues. Malignant tumors of the breast frequently display an increased capillary network and increased permeability, and these factors contribute to the earlier and stronger contrast enhancement in breast malignancies [19]. Even though MRI has an important role in the detection of breast cancers, primarily in high-risk patients, one should be aware of the fact that false-negative MR findings do occur in a small percentage of cases. MG remains the main diagnostic technique for examination of the breasts. The MR imaging technique is of complementary value in better delineation of tumor size, in recognition of additional malignant lesions, and in mammographically difficult, dense breasts [20].

Reported high sensitivity (83%-100%) of MRI for breast cancer reinforced the presumption that non-enhancing lesions on MRI were benign and did not necessitate biopsy. However, it is known that not all malignant lesions display obvious contrast enhancement and enhancement was absent in up to 12% of malignant tumors [21].

The reasons for the misdiagnosis of these lesions were technical challenges, reader perception problems secondary to masking by intensely enhancing parenchyma, small lesion size, and diffuse growth patterns. However, estimation of the types of malignant lesions that are more likely to be missed at MRI needs to be investigated in further trials.

Small tumor size and diffuse parenchymal enhancement were likely the principal reasons for these false-negative results. Although the overall sensitivity of breast MRI for cancer detection was high (96.8%), it should be emphasized that a negative MRI should not inﬂuence the management of a lesion that appears to be of concern on physical examination, mammography, or ultrasound. MRI is complementary to, but is not a replacement for, other breast imaging techniques and should not be used as the sole imaging study because, as this study shows, a small number of cancers may not be visible at MRI [21].

The detection and characterization of malignant lesions are best performed using mammography and MRI. We suggest that making an MRI evaluation before proceeding to a histopathological diagnosis for suspected and indefinite breast lesions is the most preferable approach.

Our study possesses certain limitations such as retrospective design, selection bias, and lack of a control group. The possible impacts of biomechanical, social, environmental, and ethnic confounding factors must be remembered during the extrapolation of our results to larger populations.

Ideally, the radiologists dealing with breast imaging must be familiar and experienced with not only MRI but also they must be trained especially for conventional methods such as mammography and ultrasound. These methods are complementary to each other, rather than being alternatives. It must be remembered that integrative and multi-dimensional analysis of clinical, radiological, and pathological data is mandatory to reach the accurate diagnosis soon and to initiate the appropriate treatment without delay. Rather than using MRI instead of MG+USG or biopsy, MRI findings must be comparatively evaluated with other clinical and imaging findings.

## Conclusions

Breast MRI is a sensitive imaging modality that remarkably improves screening in high-risk women. It has important functions in clinical diagnosis and staging, affecting patient management. Nevertheless, it is not a fully perfect diagnostic tool since some breast tumors may be missed and some benign lesions may be misdiagnosed as malignant. Therefore, clinical and other imaging findings from other modalities, such as mammography and USG, must be reviewed. These drawbacks must be known and shared with the patient before the performance of a breast MRI. In conclusion, dynamic MRI is a useful and reliable method for imaging breast neoplasms.
